# Inhalation Techniques Used in Patients with Respiratory Failure Treated with Noninvasive Mechanical Ventilation

**DOI:** 10.1155/2018/8959370

**Published:** 2018-06-03

**Authors:** Patrycja Rzepka-Wrona, Szymon Skoczynski, Dawid Wrona, Adam Barczyk

**Affiliations:** ^1^Department of Pneumonology, School of Medicine in Katowice, Medical University of Silesia, Katowice, Poland; ^2^Department of Organic Chemistry, Bioorganic Chemistry and Biotechnology, Faculty of Chemistry, Silesian University of Technology, Gliwice, Poland

## Abstract

The administration of aerosolized medication is a basic therapy for patients with numerous respiratory tract diseases, including obstructive airway diseases (OADs), cystic fibrosis (CF), and infectious airway diseases. The management and care for patients requiring mechanical ventilation remains one of the greatest challenges for medical practitioners, both in intensive care units (ICUs) and pulmonology wards. Aerosol therapy is often necessary for patients receiving noninvasive ventilation (NIV), which may be stopped for the time of drug delivery and administered through a metered-dose inhaler or nebulizer in the traditional way. However, in most severe cases, this may result in rapid deterioration of the patient's clinical condition. Unfortunately, only limited number of original well-planned studies addressed this problem. Due to inconsistent information coming from small studies, there is a need for more precise data coming from large prospective real life studies on inhalation techniques in patients receiving NIV.

## 1. Introduction

Inhalation therapy remains a basic treatment option for chronic airway diseases, including chronic obstructive lung disease (COPD), asthma, and in selected cases of CF. It may be used daily, as a method of symptom control or, in case of an acute exacerbation (AE), as a life-saving therapy. There is a broad spectrum of inhaler devices used in maintenance treatment for patients with mild to moderate lung diseases, including breath-actuated inhalers (pressurized metered-dose inhalers pMDIs, dry powder inhalers DPIs) [1] and nebulizers (vibrating mesh nebulizers or jet nebulizers) [2].

As noninvasive mechanical ventilation (NIV) has recently become a treatment standard in COPD [3] and also a popular treatment option for patients with respiratory insufficiency in the course of CF [4] or asthma [5], attempts are made to couple it with aerosol therapy for patients' benefit [6, 7]. Use of corticosteroids and bronchodilators during NIV has demonstrated a clinical benefit in both asthma and COPD [8]. Administration of other inhaled drugs during NIV may also be beneficial because of their physicochemical properties; for example, medications which are cleared rapidly (e.g., prostanoids) have to be administered in high locally distributed doses (antibiotics and surfactants) or for a prolonged period of time (like mucolytics) [9]. NIV is now more frequently implemented in patients admitted to the Emergency Room (ER), diagnosed with acute or acute on chronic respiratory failure in the course of OADs, who are at high risk of death. Due to the patient's clinical condition, NIV may not be discontinued to administer aerosolized drugs in the traditional manner without significant risk of rapid deterioration. Data on aerosol therapy coupled with NIV in this special patient population is scarce, and there is a need for prospective randomized trials; therefore the aim of this review of the available literature on this topic is to help medical practitioners make optimal decisions while caring for this patient population.

## 2. Inhalation Techniques in Stable COPD Treated with Chronic NIV

Inhalation therapy for stable COPD patients treated with NIV is oriented on bronchodilation and nebulized antibiotic therapy (e.g., colistin, tobramycin, or gentamycin) [10–12] in case of clinically important purulent expectoration, like in overlapping bronchiectases. Inhalation technique in patients with severe COPD may be significantly impaired, especially in end stage disease. A significant number of those patients are incapable of inhaling drugs through standard DPIs (>30 l/min) due to severely decreased inspiratory capacity (IC) resulting from poor inspiratory muscle strength, diaphragm reposition, and secondarily impaired inspiratory flows [13]. However, nebulizers are rarely used in chronic treatment due to prolonged time needed for this form of therapy [14]. Short-acting beta-agonists (SABAs) and/or ipratropium bromide are medications which are frequently used in the treatment of acute exacerbations of chronic obstructive pulmonary disease (COPD-AEs).

Abdelrahim et al. performed an in vitro experiment on terbutaline nebulization during NIV in the context of different nebulizer types (Aeroneb Pro, vibrating mesh nebulizer, and Sidestream, jet nebulizer) and the devices' position in the circuit (either proximal, e.g., the nebulizer placed between expiration port and breathing simulator—patient model or distal to the breathing simulator) during NIV with the following settings: inspiratory (*I*) and expiratory (*E*) pressures of 20 and 5 cm H_2_O, *I* : *E* ratio 1 : 3, 15 breaths/min (BPM), and tidal volume (TV) of 500 ml. During the experiment, 5 mg terbutaline was administered, and the loss of inhaled dose was measured through the amount of drug deposited in the expiration port filter. When the proximal position is considered in case of both nebulizers, it was revealed that there was a greater fine-particle dose emitted from the vibrating mesh device due to a smaller residual volume in comparison with Sidestream nebulizer [15].

Due to frequent difficulties in synchronizing inhalation and pMDI actuation, especially in elderly patients who have low mental-state scores, hand strength, and ideomotor dyspraxia [16], there is an increasing need to implement aerosol therapies which reduce the necessity of such actuation coordination in everyday practice.

## 3. Inhalation Techniques in Patients with COPD-AEs and Acute Asthma Exacerbations (AAEs) Treated with NIV

NIV is rarely used in AAEs due to lack of consistent data reinforcing its effectiveness [17]. Due to lack of official guidelines and indications for NIV in AAEs, this form of therapy is sometimes implemented as a short-term attempt, oriented on avoiding intubation and transfer to ICU. In those rare cases, continuous NIV therapy applied during NIV treatment should be maintained until clinical improvement or deterioration is observed. In case of clinical benefit, NIV should not be interrupted for aerosol therapy; therefore, the preferred option is to connect a nebulizer to a single-limb circuit in a position as close to the patient as possible. It is indicated to adjust NIV settings in a way which may facilitate an increase in bronchial drug deposition, namely, to decrease inspiratory pressures and prolong inspiratory time for the duration of drug administration. Continuous positive airway pressure (CPAP) administered in acute respiratory failure did not shorten hospitalization or reduce mortality, however, it resulted in a reduced intubation rate but did not significantly impact arterial blood gas (ABG) or forced expiratory volume in one second (FEV_1_) values [18]. In a group of patients with stable asthma, CPAP application (10 cm H_2_O) did not impact the degree of the response to nebulized albuterol or dose responsiveness [19]. In another study, it was revealed that in a group of patients with acute respiratory failure, blood oxygenation and pulmonary functions improved extensively after CPAP implementation in comparison to standard therapy [20]. AAEs are similar to COPD exacerbations in many ways, such as an increase in both inspiratory and expiratory indexes of airway obstruction, dynamic hyperinflation, and generation of negative pleural pressure that is necessary to overcome the intrinsic positive end-expiratory pressure (iPEEP) and increased airway resistance [21]. Patients treated for AAE with albuterol administered with bilevel positive airway pressure (BiPAP) exhibited greater improvement in peak expiratory flow (PEF) values than patients treated only with albuterol administered through standard nebulization [22]. Notwithstanding this, the improvement may have mostly resulted from inspiratory positive airway pressure (IPAP) unloading the respiratory muscles and reducing hyperinflation, as PEF values are more dependent on respiratory muscles' strength than bronchial patency. Alas, this has not been yet addressed in clinical research, and prospective studies are necessary to assess the determinant factors. A patient group treated for severe asthma attack with bronchodilators was randomized to NIV administered through a nasal mask with settings as follows: spontaneous over time mode (ST), IPAP 8–15 cm H_2_O (increased gradually; if <25 BPM were achieved with lower IPAP values, then lower IPAP was maintained), *expiratory positive airway pressure* (EPAP) 3 to 5 cm H_2_O 3 h/day, or sham NIV therapy. The patients were also encouraged to breathe only through the nasal mask. 80% of NIV group achieved a ca. 50% increase in FEV_1_ compared to baseline values versus 20% in sham NIV group. Intervention group also achieved a more significant increase in FEV_1_. It has to be underlined that NIV was discontinued for nebulization [23]. A study was conducted on the effect of connecting temperature, nebulizer tubing, and breathing patterns, on mass-weighted aerodynamic particle size drug distribution. The data obtained from this study suggested that when inhaled, modifications of particle distribution occur that are related to conditions in the device tubing and may reduce the diameters of particles entering the airways [24]. In terms of nebulized corticosteroids, where micronized drug suspensions are the only delivery system, in order to enhance the drug's bioavailability, superficial fluid processing reveals a significant increase in in vitro deposition of aerosol particles [25]. Moreover, positive pressures administered during NIV cause aerosol particle diameter reduction [26], respiratory rate (RR) reduction, and TV increase, which result in drug delivery enhancement [27]. Furthermore, increased expiratory time (resulting from slower RR) may promote particle sedimentation and influence aerosol deposition patterns during exhalation [27]. Pressure support (PS) and CPAP are highly effective in terms of reducing work of breathing in patients with bronchoconstriction [28–30], which could impact the response to aerosol therapy. A randomized study on patients with acute asthma exacerbation treated with NIV (median IPAP of 12 cm H_2_O, median EPAP of 5 cm H_2_O) revealed more rapid symptoms' resolution in patients receiving NIV, along with decrease in demand for bronchodilator use [31]. A prospective randomized controlled study by Brandao et al. was designed to compare the effects of jet nebulization during spontaneous breathing and aerosol therapy (2.5 mg of fenoterol bromide rate, 0.25 mg of ipratropium bromide, and 4 mL of saline solution) administered during NIV in patients with AAE and FEV_1_ <60% of the predicted value, who were treated for acute asthmatic episode in the ER. The patients were randomized into 3 subgroups: control group who were administered bronchodilators and sham NIV therapy, study group 1 (jet nebulization and NIV with IPAP 15 cm H_2_O and EPAP 5 cm H_2_O), and study group 2 (jet nebulization and NIV with IPAP 15 cm H_2_O and EPAP 10 cm H_2_O). In both study groups, the ventilator and the nebulizer were connected through a T-tube. RR, heart rate (HR), oxygen saturation (SpO_2_), PEF, FEV_1_, forced vital capacity (FVC), and forced expiratory flow at 25–75% (FEF25–75) were recorded before and after 30 minutes of each nebulization. The second study group, with greater EPAP values, revealed an increase in PEF, FVC, FEV_1_, and FEF25–75 after 30 minutes of intervention compared to baseline values. In the first study group, there was also a significant increase in PEF. This study showed that jet nebulization administered during NIV may reduce bronchial obstruction and relieve asthma symptoms better compared to aerosol therapy during spontaneous breathing. Also the potential of greater EPAP values to reduce bronchial obstruction (reflected in greater values observed for FVC, FEV_1_, and FEF25−75% in the second study group) should be underlined in this patient population [32]. This may be attributed to the improved alveolar recruitment and the improvement in patency of peripheral airways, which may in turn result in enhanced collateral ventilation in obstructed pulmonary areas [33]. Additionally, lower PS probably resulted in laminar airflow and enhanced pulmonary deposition due to drug sedimentation in collateral airways. Ventilation of peripheral lung tissue may increase pulmonary volumes and act as mucociliary clearance mechanism [34, 35], which promotes secretion clearance [36]. As revealed in a study by Soroksky et al., airway patency maintenance may be attributed to positive pressures used in NIV [23], facilitating effective nebulization performance. Nonetheless, in intubated and mechanically ventilated patients, acute with COPD-AE exacerbation, there was no difference in bronchodilator response during pressure support ventilation (PSV) or controlled mechanical ventilation (CMV) [37]. In recapitulation, several studies imply that NIV and bronchodilator therapy provide additive benefits in patients with acute asthma or COPD exacerbations. Additional studies in larger populations are necessary to confirm these findings. In spite of lack of evidence-based prospective studies on nebulized drug administration and their effectiveness in patients with severe COPD-AEs and AAEs, there is a strong impression that nebulization coupled with NIV will be more effective in patients with most pronounced bronchial obstruction. It also has to be acknowledged in asthma and COPD guidelines. NIV is much less likely to succeed in the treatment of acute asthmatic state than NIV in course of COPD-AE. This may also influence overall treatment outcomes with much poorer results in real life asthma therapy.

## 4. Inhalation Therapy with Saline Solutions in CF and COPD Treated with NIV

Nebulization with concentrated saline solutions is often used in the treatment of COPD [38] and CF [39] with large amount of dense sputum. Generally, 0.9% NaCl is used, especially in patients with a tendency for bronchoconstriction. It is rather recommended to start with 3.5% hypertonic saline and then proceed with more concentrated solutions, up to 7% [40]. This method oriented on increasing sputum clearance is often ineffective in patients with CF. Protocols based on recombinant human deoxyribonuclease followed by hypertonic saline or nebulized antibiotics such as tobramycin are implemented with these patients. Antibiotics which are typically administered intravenously may be also administered through nebulization, often as maintenance therapy [41]. Nebulization therapy is in that case followed by chest physiotherapy [42].

## 5. CF Treatment with Inhalation and NIV

CF is characterized by bronchial wall damage and thick phlegm retention leading to bronchiectasis development. These pathophysiological changes may also be spotted in severe COPD. Routine use of bronchodilators is not recommended for CF patients [43]. Application of NIV in CF patient may be also required in end stage disease with respiratory failure, especially in patients on lung transplantation list.

In a study by Fauroux et al., an evaluation of the efficacy of PSV in enhancing pulmonary deposition was conducted. Pulmonary deposition of radiolabeled drug (185 MBq of 99 m Tc phytates in 4 ml of 0.9% NaCl) was evaluated in case of aerosol produced by nebulization coupled with NIV and nebulization alone [44]. It was revealed that total pulmonary deposition was significantly improved by nebulization coupled with NIV, which was reflected in the difference in the total radioactivity count in the lungs after nebulization alone and nebulization coupled with NIV. The radioactivity count increased by ca. 30% after NIV plus nebulization intervention. The aforementioned difference cannot be in this case attributed to difference in aerosol particle size [45], as both nebulizers in the control and study group produced aerosol particles of similar diameters. As a consequence, the difference in radioactivity count can be attributed to PSV. NIV administration resulted in change in breathing pattern. In a previous study by Fauroux et al. [46], an increase in TV and decrease in RR was revealed in children with CF treated with IPAP of 12 cm H_2_O. It was found that the larger the PS, the more significant the increase in TV and the greater the decrease in RR. Also an increase in peripheral pulmonary aerosol deposition was reported in another group of patients with stable CF when a jet nebulizer was used along with positive expiratory pressure (PEP) [47]. There are no consistent data on NIV in CF with respiratory failure [48, 49], and response to bronchodilators was not evaluated in these studies.

It was revealed that PSV implemented with CF patients during chest physiotherapy prevented oxygen desaturation. Also a decrease in RR and increase in TV and minute ventilation was observed in these patients [46]. Administration of antibiotics through nebulization is a well-established treatment method in CF, but it is also effectively used in the treatment of post-lung transplant patients and in selected patients with severe COPD and bronchial Gram-negative colonization. Due to increasing interest in potential benefits to treat respiratory tract infections in mechanically ventilated patients, a consensus statement of the European Society of Clinical Microbiology and Infectious Diseases was released and following recommendations were issued. Vibrating mesh nebulizers have advantage over ultrasonic or jet nebulizers. In order to limit tracheobronchial or circuit deposition and decrease turbulence, it is recommended to use either respiratory circuits with smooth inner surface and without sharp angles or to use volume controlled mode of ventilation during nebulization with constant inspiratory flow, TV 8 mL/kg per ideal body weight, respiratory frequency 12 to 15 BPM, *I* : *E* ratio 1 : 2, inspiratory pause 20%, and positive end-expiratory pressure (PEEP) range from 5 to 10 cm H_2_O. Also, to avoid patient's flow triggering and episodes of peak decelerating inspiratory flow in case of lack of patient-ventilator coordination, administration of a short-acting sedative agent is recommended. A filter should be placed on the expiratory limb to protect the device from pathogen contamination. A heat and moisture exchanger or humidifier should be stopped during aerosol administration to avoid a massive loss of aerosol through trapping or condensation [50].

## 6. Factors Influencing Efficiency of Aerosol Therapy during NIV

Numerous factors influence the efficiency of aerosol therapy in NIV-ventilated individuals: firstly, the type of ventilator, ventilation mode, interface type, and circuit conditions; secondly, medication related factors, like particle diameter; and last, but not least, also patient-related factors impact the efficiency of drug delivery. These include breathing parameters, tolerability of particular mask/interface type, underlying diagnosis as indication for NIV, synchronization of inspiration with drug flow, severity of airway obstruction, iPEEP presence, patient-NIV synchrony, [51] and excessive leakage probably as the most important factors. Ventilation modes have impact on drug delivery due to diverse pressure settings or airflow rates. PS minimizes inspiratory effort, reduces RR, and increases TV and minute ventilation, thus improving ABG values. It also minimizes areas of atelectasis thank to PEEP and prevents small airways from closing, which may result in more uniform drug deposition [52]. Circuit humidity may increase the size of aerosol particles which results in increased impaction losses and reduction of aerosol delivery compared to a dry circuit [53].

During NIV, in contrast to invasive ventilation, air is additionally humidified during passage through nasal cavity. Many factors impact air humidification: the use of external humidifier [54], air leak [55], air temperature, breathing pattern (through the mouth or nose), and gas flow rate [54]. If big airflows resulting from high pressures are used, they may result in increased nasal resistance and bronchial hyperresponsiveness induction [55]. The increase in airway resistance reverses the beneficial effects of bronchodilator use. Heliox, an 80/20 mixture of helium and oxygen, due to lower gas density makes airflow more laminar, which reduces work of breathing in AEs of COPD therefore improving therapy tolerance [56] In healthy individuals, this gas mixture increases aerosol deposition in peripheral lung tissue, reducing its deposition in the upper airways. Use of heliox reduces drug deposition in endotracheal tube or ventilator circuit [57]. In a randomized double-blinded study, Alcoforado et al. have evaluated the effect of oxygen and heliox administered either with or without PEEP on pulmonary function and deposition of radiolabeled aerosols (fenoterol and ipratropium bromide in saline solution) in 32 stable asthmatics diagnosed with moderate to severe asthma. It was revealed that pulmonary deposition was enhanced in subgroups where PEEP was administered, regardless of gas used in nebulization process. However, medication response reflected in the increase in FEV_1_, and IC was most significant in heliox plus PEEP subgroup, in comparison to oxygen and heliox alone or oxygen plus PEEP groups [58]. The physical characteristics of heliox, which facilitate laminar airflow, generating longer expiration time, may explain the increase in IC. Prolonged expiration time allows hyperinflation reduction, thereby increasing IC. Furthermore, PEEP application during exercise helps to reduce pulmonary hyperinflation and prolong exercise [59]. Further studies are needed to explain the increase in pulmonary function in heliox plus PEEP group, compared to oxygen plus PEEP group. Heliox itself does not act as a bronchodilator, in spite of its physical characteristics. However, as mentioned above, it is possible that its high viscosity and low density (compared to oxygen) facilitate less turbulent airflow. Use of nasal masks compared to mouthpiece-based inhalation may significantly reduce pulmonary aerosol deposition due to drug deposition in the nasal passage and may lead to an increase in air leak through open mouth. Moreover, during continuous nebulization, pulmonary drug deposition may be significantly decreased if a patient removes a mouthpiece from their mouth. Nasal masks are also ineffective in case of nares obstruction [51]. Therefore oronasal masks should be chosen for first line treatment. Face mask and nasal mask should be carefully selected and tightly adjusted to avoid drug deposition in the eyes' area or aerosol leak. Nose-to-lung aerosol delivery is still a novelty in the field of aerosol therapy. Its use is limited due to significant extrathoracic particle deposition. Excipient-enhanced growth formulated drugs are generated by a DPI; a device adapted so that a patient receiving respiratory therapy through high flow nasal cannula may be simultaneously administered aerosolized medications. Condensational growth in the airways leads to an increase in particle diameter to ca. 2 *μ*m, which resulted in intrathoracic aerosol deposition >90% [60]. In a clinical trial involving healthy volunteers, who were noninvasive ventilated, urinary excretion of amikacin 24 hours postinhalation was compared after its delivery with a vibrating mesh nebulizer linked with a single-limb circuit bilevel ventilator, using conventional continuous (Conti-Neb) and experimental inspiratory synchronized (Inspi-Neb) nebulization modes. The volunteers were assigned randomly to vibrating mesh nebulization modes: Inspi-Neb delivering the amikacin solution during inspiration and Conti-Neb, delivering the antibiotic continuously. NIV using a single-limb bilevel ventilator was performed (inspiratory positive airway pressure IPAP 12 cm H_2_O, EPAP 5 cm H_2_O). The urinary concentrations of amikacin were significantly higher after administering the medication with Inspi-Neb mode, similarly to the elimination rate constant of amikacin, which is an indirect indicator of amikacin penetration into lung tissue. This suggests that Inspi-Neb mode nebulization during NIV may improve pulmonary amikacin delivery compared to conventional continuous vibrating mesh nebulization [61]. On the other hand, it has to be acknowledged that described pressures of 12/5 cm H_2_O are rather rarely used in real life NIV therapy for COPD. Further studies are needed to describe antibiotic delivery when higher pressures leading to excessive air leaks are used, as this may result in a decrease in drug deposition. Also studies with patients with severe COPD are necessary, as turbulent airflow and uneven drug distribution may be expected in case of severe airway obstruction. Medication delivery efficiencies for aerosol therapy during NIV through the ventilation circuit range from <1 to 10% in adult and pediatric populations in vitro and even less (1–6%) in vivo, the differences being attributed to lack of exhaled fractions and lack of humidification in experimental conditions. Comparatively, a study on healthy individuals was conducted to measure central and peripheral pulmonary deposition of radiolabeled diethylenetriaminepentaacetic acid (DTPA). The lung deposition was measured to be >80%, whereas extrathoracic deposition was below 20% of the body deposition [62]. Aerosol therapy during NIV has become a common practice, especially in patients with acute onset of respiratory symptoms, due to clinical benefits and such treatment's effectiveness [63].

## 7. Device Selection

Aerosol therapy may be administered to a patient receiving NIV through pMDIs or nebulizers [64–67]. Previous findings suggested better pulmonary deposition if mesh nebulizer was used during NIV in comparison with jet nebulizer [15, 68]. The most efficient devices, in case of positioning between the leak port and the face mask, were the Aeroneb Solo and NIVO [69]. It was also revealed that nebulization during NIV is more efficient than nebulization alone [44].

## 8. Interface Selection

The choice of interface depends on the patient's underlying condition and the patient's comfort. Full face mask (covering the whole face except the ears) is an interface of choice in patients with acute hypercapnic respiratory failure who are breathing through open mouth and suffer from claustrophobia or facial skin injuries. With such abundance of facial interfaces, it must be underlined that full face masks or helmets are not suitable for aerosol therapy because of ventilator flow and the patient's exposure to aerolized medications [66]. Medication leak into the patient's eyes should also be taken into account in case of patients receiving NIV [70]. Nasal masks may be used in the patient with patent nostrils who do not tolerate oronasal or full face masks. In those cases, NIV may be conducted together with nebulization throughout standard mouthpiece. In our department, mostly oronasal especially in COPD patients receiving ipratropium bromide through nebulization or eventually nasal masks are used in case of patients with large sputum volume expectoration. Oronasal masks facilitate rapid improvement of ABG in a patient with COPD-AE and are effective if breathing through open mouth cannot be eliminated. They are also easily adjusted with head straps. Full face masks are not used for ipratropium administration, as this medication must not come in contact with the patient's eyes due to its irritancy [71]. Moreover, there were reports of acute angle closure resulting from ipratropium administration, which is of special importance in patients with glaucoma [72, 73].

## 9. Delivery Technique

Aerosolized medication delivery depends on facial interface selection, device type, leak port positioning, and nebulizer location in the circuit [74, 75]. Turbine ventilators are paired either with a single-limb circuit with exhalation port or with vented mask. The positioning of leak port is significant due to medication loss to the environment. Nebulizer location between the leak port and the patient increases medication delivery during NIV independently of the device type [15, 68, 75–77] and therefore results in best clinical effects.

pMDI's efficiency in terms of medication delivery may be comparable to nebulizer's efficiency in case of the leak port's position in the circuit [75]. There were previous reports that use of mask with leak ports reduces pulmonary deposition in comparison to masks without such a port. Due to lesser medication loss during expiration with pMDIs, delivery efficacy is greater than that of nebulizers [75]. Nebulizer positioning prior to humidifier decreased medication pulmonary deposition during NIV in pediatric lung model [76]. However, humidification during acute NIV is not routinely indicated. In a study by Hassan et al., three types of devices were examined as aerosol generators in vitro, ex vivo, and in vivo: MDI with AeroChamber-MV spacer, Aerogen Pro vibrating mesh nebulizer, and Sidestream jet nebulizer. A BiPAP type ventilator (dry single-limb circuit, fixed expiratory port) was initially set in ST mode, IPAP 20 cm H_2_O and EPAP 5 cm H_2_O and later titrated to achieve TV of 500 mL in COPD patients [6]. Comparable doses of salbutamol were administered through both nebulizers, and the nominal dose of bronchodilator in the MDI device was smaller. Salbutamol deposition was later evaluated on the basis of drug concentration in patients' urine. It was revealed, that pulmonary deposition achieved with a vibrating mesh nebulizer exceeded this achieved through a jet nebulizer. However, deposition of smaller nominal dose of salbutamol (2 mg) delivered from MDI was comparable to deposition of bigger doses from both nebulizers, which may suggest better medication delivery stress on proper delivery by health care providers [6]. Tests with radiolabeled aerosols in vivo have revealed that nebulizer type may contribute to a 4-fold difference in pulmonary deposition of the aerosolized medication [78]; therefore, physicians responsible for treatment planning and drug dosage choice should be aware of the characteristics of available equipment. Previous reports demonstrated that NIV settings also impact pulmonary deposition of aerosols. Increase in IPAP improves pulmonary deposition, and increase in EPAP results in decrease in medication delivery [74]. In a bench model study by Chatmongkolchart et al., the effect of NIV settings (increasing IPAP and EPAP levels) and nebulizer position (either at ventilator outlet or between lung model connection and leak port) on albuterol delivery during NIV was evaluated. Medication deposition in the lung model was affected by nebulizer position, RR, and ventilation settings. Distal positioning of the nebulizer and RR of 20/min were associated with greatest aerosol deposition. Aerosol delivery was enhanced with increasing IPAP levels and decreased with increasing EPAP levels [74]. Therefore, nebulizer location between the facial interface and the leak port during NIV may result in delivery of approximately 25% of the nominal dose in case of high IPAP and low EPAP settings [74]; that's why it should be considered at the stage of drug dose prescription.

As stated above, clinical studies also demonstrated NIV's bronchodilator mechanism and a similar dose relationship between IPAP levels and response to bronchodilator in patients with acute asthma exacerbation [22, 32, 78].

## 10. Conclusion

Due to inconsistent data from small studies, there is a need for more precise data coming from large prospective and well-planned real life studies on nebulization techniques in patients receiving NIV. The indication for NIV coupled with aerosol therapy with most background in world literature is treatment of patients with acute and acute on chronic type-two respiratory failures in the course of COPD-AEs and AAEs. In acute setting, preferable mask type indicated in patients requiring NIV and nebulization is the oronasal mask. In patients with massive sputum expectoration, nasal mask may be used as the first line option. Nebulization combined with NIV is also indicated in CF and probably in COPD with advanced bronchiectasis, generally for antibiotic delivery and chest physiotherapy.
